# Outbreak of Middle East Respiratory Syndrome at Tertiary Care Hospital, Jeddah, Saudi Arabia, 2014

**DOI:** 10.3201/eid2205.151797

**Published:** 2016-05

**Authors:** Deborah L. Hastings, Jerome I. Tokars, Inas Zakaria A.M. Abdel Aziz, Khulud Z. Alkhaldi, Areej T. Bensadek, Basem M. Alraddadi, Hani Jokhdar, John A. Jernigan, Mohammed A. Garout, Sara M. Tomczyk, Ikwo K. Oboho, Andrew I. Geller, Nimalan Arinaminpathy, David L. Swerdlow, Tariq A. Madani

**Affiliations:** Centers for Disease Control and Prevention, Atlanta, Georgia, USA (D.L. Hastings, J.I. Tokars, J.A. Jernigan, S.M. Tomczyk, I.K. Oboho, A.I. Geller, D.L. Swerdlow);; Cairo University, Cairo, Egypt (I.Z.A.M. Abdel Aziz);; Ministry of Health, Jeddah, Saudi Arabia (K.Z. Alkhaldi, T.A. Madani);; King Fahd General Hospital, Jeddah (A.T. Bensadek, H. Jokhdar, M.A. Garout);; King Faisal Specialist Hospital and Research Center, Jeddah (B.M. Alraddadi);; Central Board of Accreditation for Healthcare Institutions, Jeddah (H. Jokhdar);; Umm Al-Qura University, Makkah, Saudi Arabia (H. Jokhdar, M.A. Garout);; International Medical Center, Jeddah (M.A. Garout);; Imperial College of London, London, UK (N. Arinaminpathy);; King Abulaziz University, Jeddah (T.A. Madani)

**Keywords:** coronavirus infections, Saudi Arabia, infection control, outbreak, transmission, nosocomial, Middle East respiratory syndrome coronavirus, viruses, MERS-CoV, vector-borne infections

## Abstract

During March–May 2014, a Middle East respiratory syndrome (MERS) outbreak occurred in Jeddah, Saudi Arabia, that included many persons who worked or received medical treatment at King Fahd General Hospital. We investigated 78 persons who had laboratory-confirmed MERS during March 2–May 10 and documented contact at this hospital. The 78 persons with MERS comprised 53 patients, 16 healthcare workers, and 9 visitors. Among the 53 patients, the most probable sites of acquisition were the emergency department (22 patients), inpatient areas (17), dialysis unit (11), and outpatient areas (3). Infection control deficiencies included limited separation of suspected MERS patients, patient crowding, and inconsistent use of infection control precautions; aggressive improvements in these deficiencies preceded a decline in cases. MERS coronavirus transmission probably was multifocal, occurring in multiple hospital settings. Continued vigilance and strict application of infection control precautions are necessary to prevent future MERS outbreaks.

## Abstract 2

Infection probably was transmitted in the emergency department, inpatient areas, and dialysis unit.

Middle East respiratory syndrome (MERS) coronavirus (CoV) is a new group C betacoronavirus first reported in a man hospitalized in Jeddah, Saudi Arabia, in June 2012 (*1*). In retrospect, MERS-CoV was found to have caused a respiratory illness cluster in April 2012 in Zarqa, Jordan (*2*). As of January 4, 2016, a total of 1,625 laboratory-confirmed MERS cases, 586 fatal, have been reported from 26 countries; >85% have been reported from Saudi Arabia (*3*). MERS-CoV infection is characterized by fever, cough, and dyspnea, and severity ranges from mild illness to acute respiratory distress, organ failure, and death; the case-fatality rate is ≈36% (*3*–*7*). Most documented symptomatic infections have resulted from human-to-human transmission (*6*). Transmission among healthcare workers (HCWs), hospital inpatients, dialysis patients, and families has been implicated in previous clusters (*2*–*5*,*8*,*9*). MERS-CoV, like other CoVs, is thought to spread through respiratory secretions; however, the precise ways the virus spreads are not well understood (*10*). The role of contact with surfaces contaminated by respiratory secretions (*5*,*11*,*12*) is unclear. Modeling done during other outbreaks estimated the incubation period to be 5.2–6.8 days (*5*,*13*,*14*).

During March 2014, the Saudi Arabia Ministry of Health (MOH) reported to the World Health Organization an increase in MERS cases in the Jeddah area (*7*). Genetic typing suggested this outbreak was caused by transmission of a single viral subtype (*15*); the reason for the increase in cases was unclear (*3*). Subsequent investigation showed that, among symptomatic patients, 21% were HCWs and 88% of evaluable non-HCWs had exposure to healthcare facilities in the 14 days before illness onset (*9*). King Fahd General Hospital (KFGH), an 815-bed public hospital, was the primarily affected facility (*15*). However, multiple MERS patients were treated in >1 healthcare facility, and the number of infections attributable to transmission at KFGH was uncertain. MERS-CoV spread at other healthcare facilities has been documented in dialysis units (*5*); early reports indicated that the KFGH dialysis unit might be the primary site of infection transmission.

Risk factors for MERS-CoV transmission at KFGH, and the efficacy of implemented control measures, were uncertain. We investigated the cluster of MERS illnesses at KFGH to determine the number of cases acquired at KFGH, identify hospital areas where transmission occurred, and assess the relationship between the implementation of infection control precautions and MERS cases.

## Methods

An assessment of the Jeddah outbreak conducted before our investigation at KFGH (*9*) produced a list of all MERS cases in Jeddah diagnosed by reverse transcription PCR (RT-PCR) conducted at the Jeddah MOH regional laboratory or Jeddah-area hospitals. This list included patient demographics, clinical data, onset date, and exposure data (including exposure to healthcare facilities) (*9*) and was used to identify ill persons with exposure to KFGH.

We defined a case as RT-PCR–confirmed MERS-CoV infection in a symptomatic person during March 2–May 10, 2014, who was exposed to KFGH as a HCW, patient, or visitor. We selected this study period to capture all persons with exposure to KFGH in the 14 days before illness onset. We categorized cases as potentially KFGH-acquired if affected persons had known KFGH contact before MERS onset and not KFGH-acquired if their first contact with KFGH followed MERS onset. We used KFGH electronic records to determine admission and discharge dates, inpatient rooms and beds occupied, and demographics for emergency department (ED) patients and inpatients. We assumed an incubation period of 2–14 days (*5*) and that patients were potentially infectious for 14 days after the onset date. To track the source of infection in individual patients, we looked for instances where an infectious inpatient was in the same room on the same date as a susceptible patient during the 2–14 days before MERS onset in the susceptible patient. We interviewed administrative, infection control, and clinical personnel at KFGH to review infection control recommendations and conducted a facility walk-through to evaluate infection control measures.

### Retrospective Cohort Study

To determine setting-specific MERS acquisition rates at KFGH, we conducted a retrospective cohort study among KFGH patients treated in the ED, inpatient areas, or dialysis unit. The cohort comprised all patients who did not have symptomatic MERS when first treated in 1 of these 3 settings. For ED and inpatient areas, we counted patient-days from the first date of treatment until MERS symptom onset, discharge, or study period end; for the dialysis unit, we estimated patient-days as explained in the Hemodialysis Unit subsection. A cohort study case was defined as onset of RT-PCR–confirmed MERS-CoV infection within 2–14 days of treatment in >1 of these 3 settings. We excluded 1 patient who had symptom onset the day after admission because the incubation period would have been <2 days after first exposure to KFGH. For patients treated in >1 of the 3 settings, we determined the most probable setting and week of acquisition based on the assumption that the incubation period followed a log-normal distribution with mean 5.2 days and SD ±1.7 days as described by Assiri et.al. (*5*) (Tables 1, 2). Other published incubation period estimates (mean 5.5 days, SD ±2.5 days [*13*]; and mean 6.8 days, no SD provided [*14*]) were used in sensitivity analyses. We calculated incidence rates as cases per 10,000 patient-days, rate ratios, and mid–p values. To support these calculations, we used maximum-likelihood modeling to provide additional estimates of infection rates and 95% CIs (Technical Appendix).

**Table 1:** Probability of Middle Eastern respiratory syndrome coronavirus incubation periods, King Fahd General Hospital, Jeddah, Saudi Arabia, March 2–May 10, 2014*

Incubation period, d	Day probability	Cumulative probability
1	0.0010	0.0010
2	0.0349	0.0359
3	0.1141	0.1500
4	0.1605	0.3105
5	0.1600	0.4705
6	0.1358	0.6063
7	0.1060	0.7123
8	0.0792	0.7916
9	0.0578	0.8494
10	0.0417	0.8911
11	0.0299	0.9210
12	0.0215	0.9425
13	0.0154	0.9579
14	0.0111	0.9690

*Incubation periods of 1–14 days by using a log-normal distribution with mean 5.2 days and SD ±1.7 days^2^.

**Table 2:** Calculation of most probable setting of Middle East respiratory syndrome coronavirus acquisition for a patient with onset date April 2, 2014, King Fahd General Hospital, Jeddah, Saudi Arabia*

Date	Setting where treated	Incubation period, d	Probability
Mar 24	ED	9†	0.0578‡
Mar 25	ED	8	0.0396§
Mar 25	Inpatient	8	0.0396§
Mar 26	Dialysis	7	0.0530
Mar 26	Inpatient	7	0.0530
Mar 27	Inpatient	6	0.1358
Mar 28	Dialysis	5	0.0800
Mar 29	Inpatient	5	0.0800
Mar 29	Dialysis	4	0.0803
Mar 29	Inpatient	4	0.0803
Mar 30	Inpatient	3	0.1141

**Figure 1:**
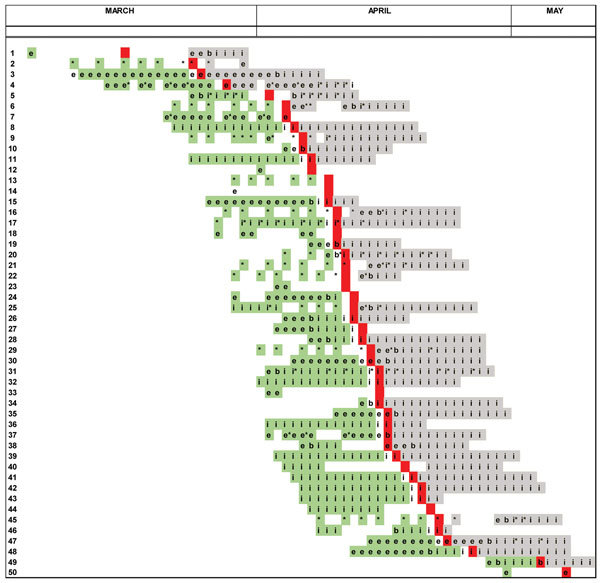
Location of cohort study patients in the 14 days before Middle East respiratory syndrome symptom onset, the day of onset, and 14 days after onset, King Fahd General Hospital, Jeddah, Saudi Arabia, March 2–May 10, 2014. Green indicates the 2–14 days before symptom onset (susceptible period); red indicates the day of onset; gray indicates the 14 days after onset (infectious period). e, emergency department; i, inpatient area; b, emergency department and inpatient areas; * indicates dialysis unit.

*Data are from patient 5 (Figure 1). The total probability is 0.50 for inpatient, 0.21 for the dialysis unit, and 0.10 for ED, so acquisition most probably occurred in the inpatient areas. ED, emergency department. †Represents the incubation period if date of disease acquisition was March 24 and disease onset was April 2. ‡Represents the probability of an incubation period of 9 days (Table 1). §Patient was treated in 2 settings on March 25, so probability is half of the probability for each setting.

### Hemodialysis Unit

From dialysis unit sign-in logs, we determined the monthly number of patients dialyzed in each of the unit’s 4 sections. Assuming each patient was dialyzed 3 times per week (standard practice), we used a linear regression model to estimate the number of patients dialyzed each day. We obtained dialysis dates and beds used by MERS patients from dialysis unit records; for days when this information was unavailable, we assigned the patients to the section where their dialysis usually occurred.

### Statistical Analyses and Ethics Considerations

For statistical analyses, we used Excel (Microsoft Corporation, Redmond, WA, USA), SAS version 9.3 (SAS, Incorporated, Cary, NC, USA), Epi Info 7 (http://wwwn.cdc.gov/epiinfo/), and OpenEpi version 3.03a (http://www.openepi.com). This investigation was reviewed by the Centers for Disease Control and Prevention and MOH and was determined to be a nonresearch public health response and not subject to Institutional Board Review.

## Results

### Patient Characteristics

We identified 130 symptomatic MERS patients who had contact with KFGH. Of these, 52 were not KFGH-acquired and 78 (60%) were potentially KFGH-acquired. The 78 comprised 50 cohort study patients (i.e., treated in >1 of the ED, inpatient, or dialysis settings) as well as 16 HCWs, 9 hospital visitors, and 3 outpatients (Table 3). Median age was lower in HCWs and visitors (both 47 years) than in cohort study patients (56 years).

**Table 3:** MERS cases potentially acquired at KFGH, Jeddah, Saudi Arabia, March 2–May 10, 2014*

Category	No. (%) cases	Patient median age, y	No. % male patients
All KFGH-acquired cases	78 (100.0)	53	59 (75.6)
Acquisition category			
Cohort study case-patient†	50 (64.1)	56	37 (74.0)
Healthcare worker at KFGH	16 (20.5)	47	12 (75.0)
Visited patient treated at KFGH	9 (11.5)	47	8 (88.9)
Outpatient appointment at KFGH	3 (3.8)	59	2 (66.7)

*Cases were categorized as potentially KFGH-acquired for persons who had KFGH contact before MERS onset. KFGH, King Fahd General Hospital; MERS, Middle East respiratory syndrome. †Patients with MERS onset 2‒14 days after emergency department, inpatient, or dialysis treatment at KFGH. Includes 3 healthcare workers (1 who worked at KFGH and 2 at other hospitals) who were admitted to KFGH inpatient areas or emergency department 2‒14 days before MERS onset.

For most of the 78 KFGH-acquired cases, illness onset occurred during April 4–22, 2014 (Figure 1). The median onset date was earlier for the 78 KFGH-acquired cases (April 11) than for the 52 non–KFGH-acquired cases (April 22). Of 16 HCWs with MERS acquired at KFGH, most (10 [63%]) were physicians; the proportion of physicians with MERS infection was much higher (1%) than for other personnel categories (Table 4).

**Table 4:** Middle East respiratory syndrome coronavirus infections among employees, King Fahd General Hospital, Jeddah, Saudi Arabia, March 2–May 10, 2014

Personnel category	No. employees	No. cases (incidence*), n = 16
Physician	958	10 (1.0)
Nurse	1,260	2 (0.2)
Technician	665	0
Administrator	515	1 (0.2)
Pharmacist	21	0
Maintenance or housekeeping	1,295	1 (0.1)
Other or unknown	†	2

*Per 100 employees. †Total number of King Fahd General Hospital personnel in this category is unknown.

### Retrospective Cohort Study

**Figure 2:**
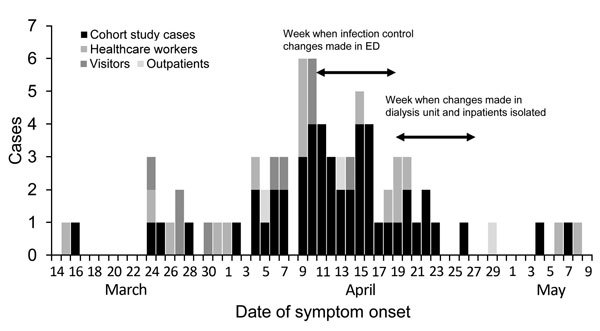
Middle East respiratory syndrome cases acquired at King Fahd General Hospital, date of symptom onset, Jeddah, Saudi Arabia, March 2–May 10, 2014 .The 78 patients comprised 50 cohort study cases, 16 healthcare workers, 9 visitors, and 3 outpatients. ED, emergency department.

The patient cohort included 2,776 hospital inpatients and 15,218 ED patients; the number of individual dialysis patients could not be determined. Among these, we identified 50 cohort study cases; these 50 patients had a median 6.5 days (interquartile range 5.0–11.0 days) of KFGH treatment during the 2–14 days before symptom onset (when they were susceptible); 41 of the 50 continued to be treated at KFGH (for a median of 12 days) in the 14 days after symptom onset, when they could have infected other patients (Figure 2). When we looked for MERS exposures outside of KFGH among the 50, we found that 1 had an outpatient contact with a known MERS patient and 1 had been admitted to a different hospital for a non-MERS illness. When we looked for documented opportunities for patient-to-patient transmission, we found that only 2 MERS patients were known to have been in the same room as an infectious patient while hospitalized at KFGH. Thirty-two of the cohort study MERS patients were exposed to only 1 setting in the 2–14 days before symptom onset; of these, 14 (44%) were exposed only to the ED, 10 (31%) to inpatient areas, and 8 (25%) to the dialysis unit. When all 50 were categorized according to the probable site of acquisition, acquisition was attributed to the ED for 22 (44%), inpatient areas for 17 (34%), and the dialysis unit for 11 (22%). In sensitivity analyses, varying the mean incubation from 5.2 to 6.8 days, the number of cases probably acquired in the ED varied from 20 to 23 and in the inpatient area from 16 to 19; the number did not change for the dialysis unit.

**Figure 3:**
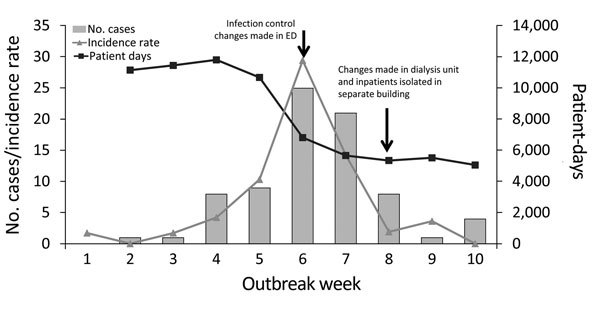
Number of Middle East respiratory syndrome cases, incidence rate, and number of patient-days of treatment at King Fahd General Hospital, Jeddah, Saudi Arabia, March 2–June 14, 2014. Incidence rate is cases per 100,000 patient-days. ED, emergency department.

Overall MERS incidence during the outbreak period was 6.1 cases/10,000 patient-days and was similar (range 5.3–6.5 cases/10,000 patient-days) for the ED, inpatient area, and dialysis unit (Table 5). Incidence was substantially higher during outbreak weeks 5–7 (March 30–April 19; rate 10.3–29.4 cases/10,000 patient-days) than during other weeks (0–4.2/10,000 patient-days) (Figure 3). The incidence rate was higher for non-Saudis than for Saudi nationals (10.1 vs. 4.5 cases/10,000 patient-days; p = 0.01) and for older persons (40–59 and >60 years, rates 19.1 and 17.4/10,000 patient-days, respectively) than younger persons (0–19 and 20–39 years, rates 2.4 and 5.9/10,000 patient-days, respectively). The illness rate was higher for male than for female persons, but the difference was not statistically significant.

**Table 5:** Retrospective cohort study of risk factors for Middle East respiratory syndrome coronavirus infection acquired at King Fahd General Hospital, Jeddah, Saudi Arabia, March 2–May 10, 2014

Population or variable	No. cases	No. patient-days	Rate*	Rate ratio	p value
Emergency department, inpatient areas, dialysis unit†	50	81,987	6.1		
Setting					
Emergency department	22	34,897	6.3	1.2	0.64
Inpatient areas	17	26,186	6.5	1.2	0.60
Dialysis unit	11	20,904	5.3	Referent	
Outbreak week: start date					
1: March 2	1	8,609	1.2	Referent	
2: March 9	0	11,136	0.0	Referent	
3: March 16	2	11,437	1.7	Referent	
4: March 23	5	11,793	4.2	4.4	0.047
5: March 30	11	10,666	10.3	10.7	<0.001
6: April 6	20	6,805	29.4	30.5	<0.001
7: April 13	8	5,654	14.2	14.7	<0.001
8: April 20	1	5,333	1.9	1.9	0.57
9: April 27	2	5,509	3.6	3.8	0.19
10: May 4	0	5,047	0.0	0.0	0.63
Emergency department, hospital inpatient areas‡	39	61,083	6.4		
Nationality					
Saudi	18	40,384	4.5	Referent	
Non-Saudi	21	20,698	10.1	2.3	0.01
Sex					
F	10	21,897	4.6	Referent	
M	29	39,151	7.4	1.6	0.18
Age group, y					
0–19	8	33,005	2.4	Referent	
20–39	10	16,888	5.9	2.4	0.064
40–59	18	9,400	19.1	7.9	<0.001
>60	3	1,725	17.4	7.2	0.016

*Per 10,000 patient-days. †For these analyses, cases were attributed to the setting or week that was most probable on the basis of incubation period (Tables 1, 2); p values do not reflect uncertainty caused by these attributions. ‡Denominator data for dialysis patients was not available; dialysis patients are not included in these analyses.

Per 10,000 patient-days, the maximum-likelihood model estimates of incidence rates were 6.4 (95% CI 3.0–9.8) for the ED, 6.2 (95% CI 2.3–10.1) for inpatient areas, and 5.5 (95% CI 1.5–9.5) for the dialysis unit; by using dialysis as the reference category, risk ratios were 1.16 (95% CI 0.45–4.45) for the ED and 1.14 (95% CI 0.36–4.29) for inpatient ares (online Technical Appendix). These values were similar to, and confirmed, results obtained by using the most probable site of acquisition.

### Hemodialysis Unit Study

Of the 50 cohort study case-patients, 22 were dialysis patients. Of the 22, the most likely site of acquisition was the dialysis unit for 11, the ED for 7, and inpatient areas for 4. Three of the 11 with dialysis unit acquisition had been dialyzed 2–14 days before their MERS onset in the same dialysis unit section and same shift as an infectious patient. For the 11 with dialysis unit acquisition, the patient-day incidence was not significantly higher in any of the 4 dialysis unit sections than in others (data not shown).

### Infection Control Evaluation

Early in the study period, ED patients were treated in 1 of 5 rooms, according to illness acuity. The ED was typically crowded, with patient gurneys nearly touching one another; no system for triaging and isolating patients with respiratory diseases was available, and patients often remained in the ED for multiple days under the care of admitting service physicians. During epidemic week 6 (April 6–12), KFGH implemented a system of aggressive ED triaging to separate patients with respiratory symptoms from other ED patients. Patient census in the ED decreased (Figure 3), enabling increased separation of patient gurneys.

The HCWs in the ED and intensive care unit were screened for MERS-CoV with RT-PCR (number screened unknown). Fourteen were positive and isolated at home; 13 of these remained asymptomatic and returned to work when they became RT-PCR negative (these 13 were not included as cases because they were asymptomatic). One positive HCW became ill while in home isolation, was hospitalized, and is included among the 78 cases.

Before the outbreak, MERS inpatients were isolated or cohorted in standard patient rooms, but infection control adherence was reportedly incomplete. Starting in week 8 (April 20–26), inpatients with known or suspected MERS were treated in 1 of 17 single-patient negative-pressure isolation rooms in a new hospital building; visitors were not allowed in patient rooms. Also during week 8, KFGH was designated a MERS treatment facility and began accepting transfers of MERS patients from other Jeddah hospitals. Starting May 9, KFGH leadership made additional changes, including implementation of a new MOH infection prevention and control guideline and management algorithm (*16*,*17*) that included a hospital-based clinical guideline with detailed procedures for treating MERS patients. Transport pathways, elevators, a radiograph room, and a resuscitation room were designated for MERS patient use.

Before the outbreak, dialysis was performed 24 hours/day during 6 days/week. During week 8 (April 20–26), after a visit from Saudi MOH officials, changes were made: dialysis was reduced from 4 to 3 shifts per day, space between patients was increased to a minimum of 1.2 m, and use of personal protective equipment (gowns, gloves, and surgical masks) among HCWs was required. None of the dialysis unit HCWs developed MERS during the outbreak period.

## Discussion

Our results suggest that 78 persons (53 patients, 16 HCWs, 9 visitors) became symptomatically infected with MERS at KFGH during spring 2014. Among the patients, infection was acquired in multiple settings, including the ED (22 cases), inpatient areas (17 cases), outpatient dialysis unit (11 cases), and other outpatient areas (3 cases). We were able to directly track transmission (i.e., an infectious and a susceptible patient in the same room) only to 5 cases: 2 in inpatient areas and 3 in the dialysis unit. However, most of these persons had several days (median 6.5) of treatment in KFGH before MERS onset (Figure 1), strongly suggesting that they acquired MERS at KFGH. In addition, we were able to determine setting-specific infection rates and found that they were similar in the ED, inpatient areas, and dialysis unit, indicating that risk for acquisition was widespread. Treatment in >1 setting of many of these patients complicated determination of these setting-specific rates. However, the 2 methods we used to overcome this problem, most likely site of acquisition and maximum-likelihood modeling, provided good agreement.

In previous MERS outbreaks, dialysis units have been implicated as a focus of disease transmission. At KFGH the patient-day risk for transmission in the dialysis unit was no greater than in the KFGH ED and inpatient areas; only 11 of the 22 cohort patients receiving dialysis were believed to have acquired MERS in the dialysis unit.

Although MERS incidence rates were similar in the ED, inpatient areas, and dialysis unit, the ED was the most frequently implicated in disease acquisition among patients. Contributing factors probably included lack of triage, lack of isolation for patients with respiratory symptoms, extended stays for certain patients, and close proximity among patients. The KFGH outbreak peaked during week 6 and decreased sharply after week 7 (Figures 2, 3) after infection control improvements in the ED during week 6. During week 8, infection control was improved in the dialysis unit, and a MERS unit was established in a separate building. These improvements, in combination with decreased patient volume by week 6, probably led to decreased transmission.

The median onset date for KFGH-acquired MERS-CoV infection (April 11) was ≈2 weeks before that of cases that were not KFGH-acquired (April 22) and 1 week before all symptomatic cases in Jeddah (US Centers for Disease Control and Prevention, unpub. data). Thus, as indicated in a recent laboratory-based study (*15*), KFGH appears to have been an early focus of transmission in the Jeddah outbreak. Our cohort study reported higher incidence among older patients at KFGH, suggesting that clinically recognized disease did not develop in certain younger patients who were exposed to MERS-CoV infection at KFGH. Although we did not study HCW-to-patient transmission, asymptomatic HCWs were documented and might have contributed to disease transmission. The potential for transmission by asymptomatic persons needs further investigation.

Limitations to this study include incomplete clinical data for patients and for HCWs. In certain instances, we had to use clinical judgement to estimate disease onset dates. No records were kept on ED rooms in which patients were treated. Although we attempted to identify all non-KFGH healthcare exposures, certain exposures might not have been identified. We assumed that patients treated at KFGH at any time during the 2–14 days before illness onset had potentially KFGH-acquired disease. Records were not uniformly available when patients from other dialysis units or from the ED were dialyzed temporarily at KFGH; our results might not reflect disease transmitted from, or acquired by, these patients. The number of dialysis patient-days of treatment could only be estimated. Finally, data on genetic typing of MERS-CoV viruses from KFGH cases that could have helped define chains of transmission was not available to us.

In conclusion, the MERS outbreak at KFGH affected patients and HCWs. Illness most likely was transmitted in multiple settings, making it difficult to track disease from patient-to-patient. The contribution to MERS-CoV transmission at KFGH by asymptomatic persons is uncertain; transmission by asymptomatic persons is an area that needs further study. Heightened awareness of MERS, aggressive triaging of patients, prompt isolation, and strict infection control measures were associated with a rapid decrease in transmission. Continued vigilance and consistent adherence to infection control precautions is necessary to prevent future healthcare-acquired MERS outbreaks.

Technical Appendix Maximum-likelihood modeling to provide additional estimates of MERS-CoV infection rates.

## Footnotes

2 Current affiliation: International Medical Center, Jeddah, Saudi Arabia.
