# Supplementary material for: Outbreak of Middle East Respiratory Syndrome at Tertiary Care Hospital, Jeddah, Saudi Arabia, 2014
Source: Emerg Infect Dis. 2016 May;22(5):794–801. doi: 10.3201/eid2205.151797 (PMC4861521; doi:10.3201/eid2205.151797)
Supplement: Technical Appendix — Maximum-likelihood modeling to provide additional estimates of MERS-CoV infection rates. [file 15-1797-Techapp-s1.pdf]

Article DOI: http://dx.doi.org/10.3201/eid2205.151797

Outbreak of Middle East Respiratory Syndrome at Tertiary Care Hospital, Jeddah, Saudi Arabia, 2014

Technical Appendix

The daily risk for infection in each of the 3 settings was estimated by maximizing the log-likelihood of observing the given onset dates, incorporating the patient exposure to each setting and the incubation period distribution (Table 1). For uncertainty intervals, we estimated the covariance as the inverse of the negative Hessian matrix, at the maximum log-likelihood estimate (*1*). We then used this in a multivariate normal distribution, taking 10,000 samples, calculating the relative risk with each draw, and taking the 2.5, 50, and 97.5 percentiles of the resulting values.

We denote S_i as the setting that the patient occupied on day *i*, taking values from "E," "I," or "D" (representing, respectively, the emergency department, inpatient areas, and dialysis unit). Thus, for example, a series $[S_1, S_2, S_3]$ = [E, D, D] represents a patient who was in the emergency department on day 1, then spent 2 days in a dialysis unit before symptom onset.

In each setting, we assume that a patient has a daily probability of infection of *p*(E), *p*(I), *p*(D), respectively. Further, for a patient who acquires infection on day *i*, we denote IP_{ij} as the probability that this patient subsequently shows onset on day *j*.

With S_i given by data, and IP_{ij} given by the log-normal distribution for the incubation period, we estimated the daily risks *p*(E), *p*(I), *p*(D) by a maximum-likelihood approach. In particular, for a single patient *n*, with an exposure history $S_i^{(n)}$, the probability of getting infected on day *i* and showing symptoms on day j is

$$y_{ij}^{(n)} = \left[\prod_{k<i} 1 - p\left(S_k^{(n)}\right)\right] \times p\left(S_i^{(n)}\right) \times IP_{ij}$$

That is, terms in (1 – p) are a product of all the days that this patient avoided infection, leading up to day i; the lone term in p denotes the day that this patient acquired infection on day i; and the final term represents the contribution from the incubation period.

For patient n, the likelihood of showing symptoms on day J is then given by a summation over all possible days of infection i <J, that is

$$L^{(n)} = \sum_{i<J} y_{iJ}^{(n)}$$

To find the overall log-likelihood, we calculated

$$loglihood = \left[\sum_{n} \log\left(L^{(n)}\right)\right] + \log[(1-p_E)^{m_E}] + \log[(1-p_I)^{m_I}] + \log[(1-p_D)^{m_D}]$$

where m_E, m_I, m_D are the number of patient-days spent in each of the hospital settings, that were *not* associated with infection.

To obtain point estimates for the daily risks, we maximized the log-likelihood with respect to p(E), p(I), p(D). To find the uncertainty intervals, we followed standard likelihood techniques (*1*) in calculating the inverse of the Hessian matrix at the maximum-likelihood estimate. This provides an estimate of the variance-covariance matrix; we used this in a multivariate normal distribution, taking 10,000 samples in a Monte Carlo simulation, and finding percentiles of the resulting values.

Reference

1. Rohde C. Introductory statistical inference with the likelihood function. Cham, Switzerland: Springer International Publishing; 2014.
